# The relationship of muscular endurance and coordination and dexterity with behavioral and neuroelectric indices of attention in preschool children

**DOI:** 10.1038/s41598-022-11161-4

**Published:** 2022-04-29

**Authors:** Shih-Chun Kao, Yu-Jung Tsai, Shu-Shih Hsieh, I-Fan Chen, Sara Schmitt, Tsung-Min Hung

**Affiliations:** 1 Department of Health and Kinesiology, Purdue University, West Lafayette, USA; 2 Department of Physical Education, National Taiwan Normal University, Taipei, Taiwan; 3 Department of Psychology, Kingston University, Kingston upon Thames, UK; 4 Department of Human Development and Family Studies, Purdue University, West Lafayette, USA

**Keywords:** Psychology, Cognitive neuroscience

## Abstract

This study investigated the associations of non-aerobic fitness (NAF) and motor competence (MC) with attention in 4–6 year-old preschoolers. The allocation of attentional resources and speed of stimulus categorization were examined using the amplitude and latency of P3 of event-related potentials respectively, while cortical activation related to general attention and task-specific discriminative processes were examined using event-related desynchronization (ERD) at lower (8–10 Hz) and upper (10–12 Hz) alpha frequencies, respectively. Seventy-six preschoolers completed NAF (muscular power, muscular endurance, flexibility, balance) and MC (coordination and dexterity, ball skills, agility and balance) test batteries. Electroencephalogram was recorded while participants performed an auditory oddball task. After controlling for age and MC, muscular endurance was positively related to P3 amplitude. MC and its coordination and dexterity sub-component were positively related to task performance, with higher levels of coordination and dexterity showing an additional association with greater upper alpha ERD between 700 and 1000 ms following stimulus onset after controlling for age and NAF. These findings suggest relationships of NAF and MC with early childhood neurocognitive function. Specifically, muscular endurance is related to the neuroinhibition in facilitating effective allocation of attentional resources to stimulus evaluation while coordination and dexterity are related to cortical activation underlying strategic attentional preparation for subsequent stimulus evaluation.

## Introduction

Early childhood is a time of rapid postnatal brain maturation concurrent with acquisition of cognitive abilities relying on the attention network^1^. The attention network plays an essential role in prioritizing sensory input by focusing on a modality or location as well as exerting top-down controlled processes to inhibit or facilitate the allocation of attention toward behaviorally relevant events^2^. During the preschool years, these attention systems are vital for development and school readiness because they support self-regulatory processes of cognitive and emotional reactivity to the environment^3^. Further, the inter-individual variability in these cognitive abilities has been associated with early development of physical abilities such as physical fitness^4^ and motor skills^5^.

Physical fitness refers to a set of attributes that reflect health- and skill-related components necessary to carry out daily tasks and engagement in physical activity^6^. Aerobic fitness is the ability of the circulatory and respiratory systems to supply oxygen during physical activity^6^ and has a positive relationship with cognitive health^7^ likely through its mechanistic role in the cerebral oxygenation^8^ in support of brain networks responsible for attention and executive function^9^. During preschool years, aerobic fitness is associated with better attention^10^, attentional inhibition^11,12^, and perceptual/conceptual skills^13^.

Non-aerobic fitness (NAF) has also been shown to correlate with early childhood cognition. Muscular fitness, which can be separated into power and endurance to index the maximum muscle-generated force and the ability to sustain repeated muscle contractions^6^, was also related to better attention^14^. Following 8–10 weeks of exercise designed to increase muscle fitness, preschoolers improved performance during tasks requiring attention^15^ and attentional inhibition^16^. Although less studied, flexibility reflects the range of motion available at a joint^6^ and its improvement has been linked to increased attentional inhibition^16^. Relatedly, balance indexes a skill-related ability to maintain static or dynamic equilibrium^6^. This fitness component has also been positively associated with general cognitive abilities^17^, working memory^10^, and spatial/proportional reasoning^18^ in preschoolers.

Motor competence (MC) refers to the foundational ability underlying movement skills that enable engagement in physical activity and positively contributes to not only physical fitness^19^ but also cognitive development^20–22^. The beneficial associations of MC with behavioral outcomes of attention and executive function have been observed in preschool children^14,23^. Further, a systematic review indicated positive relations between motor and cognitive skills, suggesting the feasibility of complex motor intervention programs to stimulate both motor and higher-order cognitive skills during early childhood^24^. Indeed, recent intervention programs that targeted MC showed improvements in executive function and numeracy skills in 3–6 year-old children^25,26^. Further, research comparing different sub-components of MC has shown that fine motor skills (i.e., small and precise movements such as holding and moving a small object) may be a more sensitive correlate of cognitive function^27^ and later academic achievement^28^ compared with gross motor skills (i.e., whole-body movements involving larger muscle groups such as jumping and running). Taken together, the positive associations of physical fitness and MC with cognitive abilities in preschool children may be behavioral manifestations of enhanced underlying information processing and superior brain development. However, this speculation on the potential neurocognitive benefits of physical fitness and MC beyond behavioral task performance has remained largely unknown in this preschool population and needs to be empirically determined^29^.

Deriving from electroencephalogram (EEG), P3 (also known as P300) is a positive ongoing deflection of brain potential peaking between 300 and 800 ms following stimulus onset and indicates the updating of mental representation as a result of top-down controlled attention reacting to a bottom-up stimulus^30^. P3 has widespread and synchronous neural generators centered at the cingulate cortex linking frontal-mediated attention and updating to memory storage operated in the temporal/parietal brain areas^31–33^. The neural substrates of P3 correspond with the executive attentional networks^2,34^, which coordinate bottom-up vigilance and top-down control over monitoring for, resolution of, and responding to conflicts^3^. P3 can be elicited during tasks requiring attentional and executive control processes, with increasing amplitude reflecting greater neural inhibition to irrelevant processing while allocating attentional resources to the task at hand and decreasing latency reflecting a faster speed of stimulus categorization^30^. In addition to the well-documented correlations of these P3 indices with actual performance during attention tasks and clinical outcomes, P3 is a neuroelectric marker that reflects childhood brain development^35^. From early childhood to adolescence, P3 amplitude increases and P3 latency decreases^35,36^, reflecting the maturing functional brain mechanism in response to environmental stimuli through more effective neuroinhibition^30^.

During preadolescence, greater aerobic fitness has been repeatedly associated with increased P3 amplitude and/or decreased P3 latency along with superior cognitive performance during tasks requiring attention and executive control^4,37–39^. This cross-sectional association has also been confirmed by increases in both aerobic fitness and P3 amplitude following a 9-month physical activity intervention^40^. Although less studied, similar beneficial associations of muscular fitness^41,42^ and MC^43^ with P3 have been observed. These beneficial associations may reflect more effective neuroinhibition in facilitating increased allocation of attentional resources to and enhanced speed for evaluating task-relevant stimulus^44^ as well as more matured brain function^35^ in children who are more physically fit or competent in motor skills. Recent research has extended this association to preschool children, with preliminary evidence showing a positive correlation between distance walked in a 6-min walking test, as an estimate of aerobic fitness, with P3 amplitude during an auditory stimulus discrimination task^12^, suggesting the potential importance of aerobic fitness to neuroelectric function during early childhood. However, the existing literature in preschool children lacks an understanding of P3 in relation to NAF domains and MC, which are closely related to early childhood physical health outcomes^20,45^ and neurocognitive function^21^.

Unlike P3 reflecting neuroelectric signals summed over a range of oscillatory frequencies, alpha event-related synchronization (ERS) and desynchronization (ERD) refer to task-related increases or decreases, respectively, in electrophysiological oscillations at 8–12 Hz frequencies^46^. Because alpha ERS and ERD provide an approximated synchrony and asynchrony of the underlying neuronal populations^47^ in support of functional inhibition and disinhibition of task-relevant activation^48^, these complementary alpha-specific measures enable the examination on dynamic changes of cortical activation during cognitive tasks. Further, the alpha frequency band can be separated into lower alpha (8–10 Hz) and upper alpha (10–12 Hz) bands, which respectively reflects general attention and task-specific processes (i.e., discriminative, semantic) related to cognitive performance^49^. Although alpha ERS can be observed during the early stage of stimulus evaluation, a more pronounced ERD at both lower and upper alpha bands in the parietal region (i.e., PZ electrode site) follows^49,50^ and indexes increased cortical activation in support of attention and task-specific processes^46^. Further, research has demonstrated a temporal relation between alpha ERD and P3 during an auditory oddball task, with target-eliciting parietal lower and upper alpha ERD following P3^50^. Given that increased alpha ERD has been associated with superior cognitive performance^49,50^, these findings were interpreted to suggest that the extent of available attentional resources for the discrimination process as a result of inhibiting task-irrelevant processing may be related to the following reset of alpha oscillation, which indicates the release from inhibition in preparation for subsequent task performance^46,50,51^. Further, parietal alpha ERD was found to be a neuroelectric representation of brain activation involving the fronto-parieto-occipital attention network^52–54^, which is central to the development of the attention network during preschool years^2^. Despite the potential role of alpha ERD to the attentional processes underlying superior cognitive performance and the functional manifestation of the attention network, its association with physical abilities such as NAF, MC, and their sub-components during early childhood remains unknown.

The purpose of this study was to investigate the association of early childhood NAF, MC, and their sub-components with behavioral attention performance and underlying neuroelectric brain functioning using P3-ERP and alpha ERD. These neuroelectric indices were chosen because of their functional significance to task-related attentional processes as well as brain function during early childhood. Based on the established relations of NAF and MC with P3 in older children^41–43,55^, it was hypothesized that higher levels of NAF, MC, and their sub-components would be associated with larger P3 amplitude and shorter P3 latency. Additionally, given the well-established association of alpha ERD with enhanced cognitive performance^49^, it was hypothesized that higher levels of NAF, MC, and their sub-components would be associated with greater alpha ERD. Findings consistent with our predictions would improve the understanding of the beneficial associations of physical abilities with neuroelectric mechanisms underlying superior brain function, as evidenced by faster speed of stimulus categorization, more effective attentional allocation, and increased cortical activation in support of attentional performance. Given that the Committee Scientific Report of the 2019 Physical Activity Guidelines^29^ has highlighted the lack of understanding in the relationships of early childhood physical activity with cognitive development, particularly its underlying neural adaptation, there is a critical need to begin establishing the associations of developmentally-relevant neural markers such as P3 and alpha ERD with biobehavioral correlates of physical activity. The identification of these neural correlates of cognitive performance and their relations with childhood NAF and MC will contribute to the field by informing future research determining the mechanistic role of these neuroelectric indices in the physical activity and cognition relationship during early childhood.

## Results

Participants’ demographic and NAF and MC variables as well as the behavioral and neuroelectric outcomes during the auditory oddball task are shown in Table 1. Sixteen participants were excluded due to insufficient artifact-free EEG epochs (< 15). Seventy-six participants who completed all testing and had a minimum of 15 accepted artifact-free EEG epochs for quantifying P3 (target: 34 ± 10; standard: 135 ± 38) and alpha ERD (target: 34 ± 10; standard: 135 ± 39) outcomes were included in the statistical analysis. The analyzed sample should allow a power = 0.87 to detect the hypothesized associations of NAF and MC with behavioral (*f*^2^ = 0.16)^14^ and neuroelectric indices of attention (*r* = 0.37)^12^.

**Table 1:** The mean and standard deviation of demographic, non-aerobic fitness, motor competence, and behavioral and neuroelectric measures.

Measure	Boys (N = 49)	Girls (N = 27)	All (N = 76)	Range
**Demographic measure**				
Age (month)	68.1 ± 3.9	68.9 ± 6.2	68.4 ± 4.8	51–84
Creativity (score)	107 ± 68	84.8 ± 65.5	99.4 ± 67.8	11–296
**Non-aerobic fitness measure**				
Stand long jump (centimeter)	101.6 ± 15.0	98.1 ± 20.1	100.4 ± 16.9	48–142
60-s sit-up (repetition)	7.2 ± 4.7	7.3 ± 4.9	7.1 ± 4.8	0–18
One-leg, eye-closed, standing on a beam (second)	3.5 ± 2.9	4.0 ± 2.6	3.7 ± 2.8	0.88–15.54
Sit-and-reach (centimeter)	22.7 ± 6.4	24.8 ± 5.6	23.4 ± 6.2	10.5–35.5
**Motor competence measure**				
Bead stringing (count)	8.7 ± 2.5	9.0 ± 1.9	8.8 ± 2.3	1–13
Target throwing (hit)	11.0 ± 3.4	9.7 ± 4.0	10.5 ± 3.5	1–17
Marble transfer (count)	31.8 ± 12.4	32.6 ± 10.8	32.1 ± 11.8	16–58
Ball striking (score)	10.6 ± 9.1	8.4 ± 9.0	9.9 ± 9.1	0–34
Basketball throwing (centimeter)	102.4 ± 44.4	91.9 ± 45.7	98.8 ± 44.8	20–200
Ball kicking (score)	12.3 ± 6.8	13.2 ± 7.6	12.7 ± 7.1	0–17
Static balance (second)	73.4 ± 63.8	119.7 ± 143.7	90.1 ± 100.4	0.4–643
Prone-stand-turn position (count)	6.9 ± 2.8	6.6 ± 2.9	6.8 ± 2.8	2–15
4-cone shuttle run (second)	7.3 ± 0.9	7.8 ± 1.1	7.5 ± 1.0	5.7–10.6
**Behavioral and neuroelectric measure**				
Hit (percent)	92.7 ± 7.3	91.1 ± 9.0	92.1 ± 7.9	65–100
FA (percent)	3.0 ± 2.5	2.5 ± 1.6	2.8 ± 2.2	0–11
*d*′	3.6 ± 0.7	3.5 ± 0.7	3.6 ± 0.7	2.1–4.8
RT (millisecond)	574 ± 88	615 ± 158	589 ± 118	380–1126
P3 amplitude (microvolt)	16.1 ± 9.2	14.8 ± 7.0	15.6 ± 8.5	− 3.7 to 34.5
P3 latency (millisecond)	644 ± 120.8	615 ± 118	633 ± 120	314–790
Lower alpha ERD (decibel)	− 1.9 ± 1.5	− 1.8 ± 1.5	− 1.67 ± 1.5	− 5.7 to 1.4
Upper alpha ERD (decibel)	− 0.9 ± 1.5	− 0.7 ± 1.7	− 0.85 ± 1.6	− 8.5 to 3.9

The creativity scores were calculated based only on 73 participants (see Supplement for more details).

### Parietal P3 and alpha ERD

**Figure 1:**
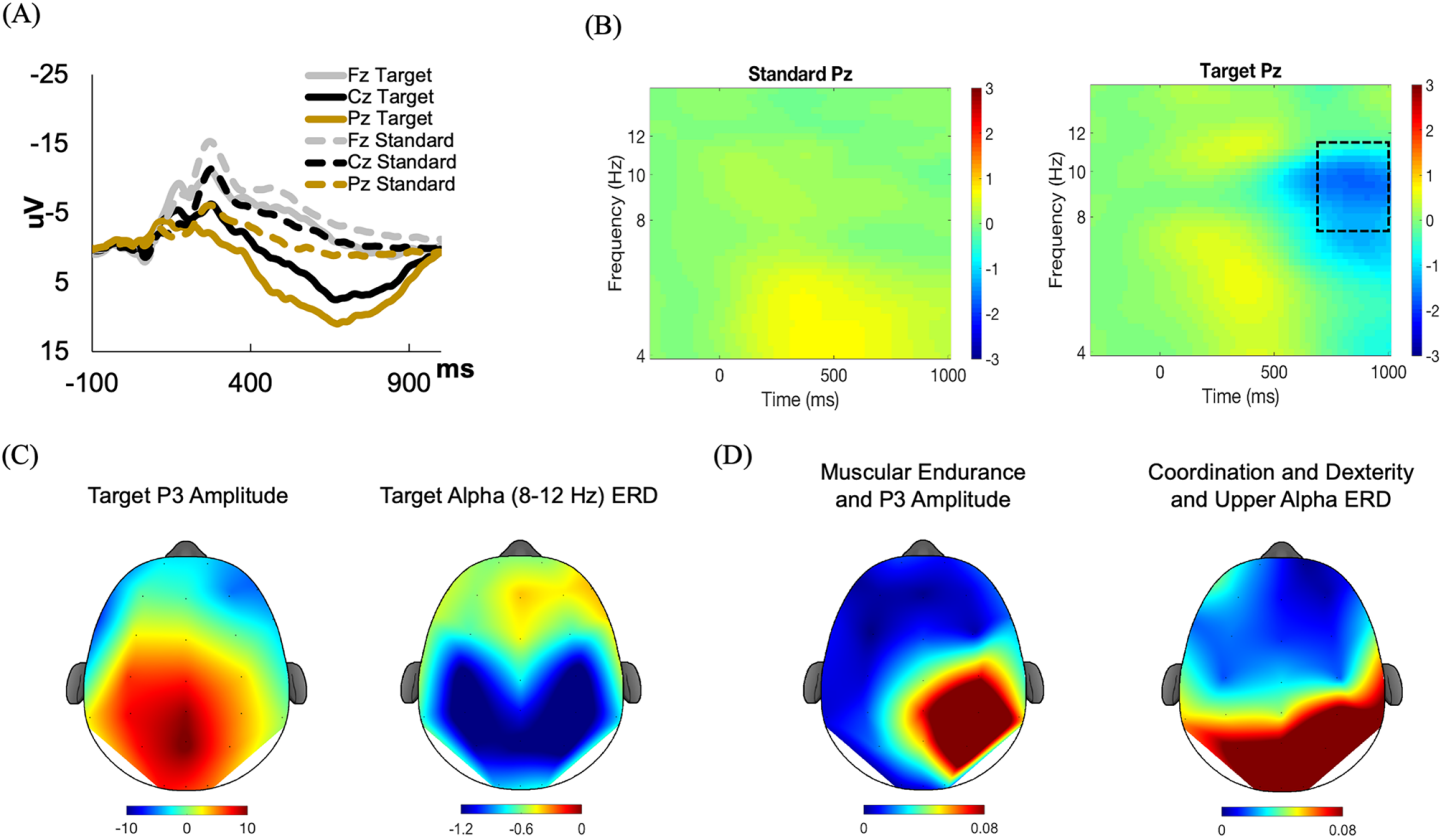
P3-ERP and alpha ERD during the auditory oddball task and their associations with muscular endurance and coordination and dexterity. (**A**) The ERP grand average waves show a P3 peaked around 650 ms after the onset of target stimulus (solid lines) at the Pz electrode (tan lines) while a weaker P3 was observed following standard stimulus (dashed lines) at Fz (gray lines) and Cz (black lines). (**B**) The time–frequency representation of oscillatory activities at Pz electrode shows a desynchronization of both lower (8–10) Hz and higher (10–12 Hz) alpha frequency from 700 to 1000 ms (dotted rectangle) following the onset of target (right) stimulus but not standard (left) stimulus. (**C**) The scalp distributions of P3 amplitude and the ERD at the alpha (8–12 Hz) band following the target stimulus were stronger at the parietal region. (**D**) The topographical distribution of the R^2^ changes for each electrode obtained from the final step of the 3-step hierarchical regression analysis on the association of muscular endurance with P3 elicited by target stimulus and the associations of coordination and dexterity with upper alpha ERD elicited by target stimulus.

A clear target P3 was observed at Pz (Fig. 1A,C). This observation was confirmed by a Stimulus × Electrode interaction, *F* = 38.2, *p* < 0.001, η_p_^2^ = 0.338, showing that the increased amplitude for target compared to standard stimuli at Pz (12.0 ± 1.0 uv) and Cz (10.6 ± 0.9 uv) were significantly greater compared to Fz (5.6 ± 0.8 uv), *p*s < 0.001. Though alpha ERS was relatively weak at the upper alpha band and absent at the lower alpha band, a clear alpha ERD in response to target stimulus onset was observed at the Pz electrode between 700 and 1000 ms, which was used to calculate the average alpha ERD for subsequent statistical analysis (Fig. 1B,C). The selection of the time–frequency representations of interest in lower and upper alpha bands at Pz was confirmed by a Stimulus × Electrode interaction, *F*s < 12.8, *p*s < 0.001, η_p_^2^ < 0.146, showing that the increased ERD from standard to target stimuli at Pz (lower: − 1.786 ± 0.184 db; upper: − 0.925 ± 0.193 db) was significantly greater compared to Cz (lower: − 1.095 ± 0.171 db; upper: − 0.457 ± 0.164 db) and Fz (lower: − 0.537 ± 0.160 db; upper: − 0.234 ± 0.162 db), *p*s < 0.001. Taken together, the observed P3 and alpha ERD specifically following the target stimulus at Pz electrode site replicated previous findings in older children and adults^37,49^, confirming the adequacy of the P3 and alpha ERD measures at Pz for subsequent analysis (see supplement 1 for detailed justification).

### Correlation analysis

As shown in Table 2, age was positively correlated with all task performance indices as well as NAF, MC, and their sub-components including muscular power, muscular endurance, coordination and dexterity, and ball skills. Sex and creativity were not correlated with any variables, except for the agility and balance sub-component score that was lower for female and less creative individuals compared with their counterparts. NAF, MC, and their sub-components including muscular endurance, muscular power, coordination and dexterity, and ball skills were related to task performance and/or neuroelectric outcomes.

**Table 2:** The correlation matrix showing correlations (*r* coefficients) of demographic variables with non-aerobic fitness, motor competence, and the behavioral and neuroelectric outcomes.

	Age	Sex	Create	NAF	MC	Power	Endur	Flex	Balan	Coordination and dexterity	Ball Skills	Agility and Balance
Age	–	0.082	− 0.105	0.272*	0.383*	0.309*	0.266*	− 0.154	0.108	0.252*	0.261*	− 0.054
Sex	–	–	− 0.161	0.069	− 0.082	− 0.094	0.006	0.165	0.071	− 0.045	− 0.081	0.303*
Create	–	–	–	− 0.113	− 0.104	0.041	− 0.061	− 0.194	− 0.008	− 0.020	0.057	− 0.281*
NAF	–	–	–	–	0.496*	0.602*	0.592*	0.463*	0.308*	0.248*	0.383*	− 0.027
MC	–	–	–	–	–	0.487*	0.434*	− 0.081	0.119	0.640*	0.693*	0.048
Hit	0.237*	− 0.096	− 0.058	0.406*	0.509*	0.336*	0.219	0.105	0.136	0.364*	0.254*	0.079
FA	− 0.261*	− 0.104	0.061	0.068	− 0.116	− 0.047	− 0.04	0.136	0.101	0.023	− 0.080	− 0.194
*d*′	0.231*	− 0.026	0.000	00.21	0.395*	0.256*	0.159	− 0.021	0.005	0.237*	0.226*	0.130
RT	− 0.385*	0.188	− 0.183	− 0.326*	− 0.465*	− 0.303*	− 0.333*	0.079	− 0.078	− 0.366*	− 0.302*	0.137
P3A	0.102	− 0.071	− 0.039	0.250*	0.227*	0.216	0.306*	0.034	− 0.096	0.214	0.122	− 0.122
P3L	− 0.068	− 0.119	− 0.106	0.002	0.019	− 0.019	0.002	0.161	− 0.120	− 0.011	0.019	0.081
Lower alpha ERD	− 0.158	− 0.083	− 0.024	− 0.202	− 0.258*	− 0.205	− 0.169	− 0.070	0.069	− 0.255*	− 0.188	0.001
Upper alpha ERD	− 0.163	0.050	0.063	− 0.277*	− 0.322*	− 0.234*	− 0.148	− 0.086	− 0.074	− 0.313*	− 0.239*	0.093

Bold values with a * denote significant correlation at *p* < 0.05. Create = creativity. NAF = non-aerobic fitness. MC = motor competence. Power = muscular power. Endur = muscular endurance, Flex = flexibility, Balan = Balance. P3A = P3 amplitude. P3L = P3 latency.

### Hierarchical regression analysis

#### Non-aerobic fitness

Some of the NAF-related correlations with neurocognitive outcomes remained significant in the 2-step hierarchical regression analysis after controlling for age (see Table 3), with muscular power positively associating with hit rate, greater muscular endurance associating with shorter RT and larger P3 amplitude, and higher NAF associating with higher hit rate, shorter RT, and decreased upper alpha (i.e., greater ERD). After further controlling for MC in the 3-step models, only a positive association between muscular endurance with P3 amplitude was observed (see Fig. 1D).

**Table 3:** The summary of the 2-step and 3-step hierarchical regression analyses using non-aerobic fitness or its subcomponent as a predictor.

	Step 1	2-Step model			3-Step model		
		Step 2			Step 3		
	*R*^2^	*△R*^2^	F	*Beta*	*△R*^2^	F	*Beta*
**Muscular power**							
Hit	0.056*	0.076*	5.59*	0.291*	0.009	8.91*	0.110
d′	0.053*	0.038	3.66*	0.205	0.004	4.82*	0.070
RT	0.148*	0.029	7.88*	− 0.180	0.001	8.77*	− 0.034
Upper alpha	0.022	0.029	1.96	− 0.178	0.007	3.04*	− 0.096
**Muscular endurance**							
RT	0.148*	0.057*	9.44*	− 0.248*	0.014	9.38*	− 0.134
P3 amplitude	0.010	0.084*	3.79*	0.300*	0.053*	2.80*	0.257*
**Non-aerobic fitness**							
Hit	0.056*	0.126*	8.12*	0.368*	0.030	9.87*	0.199
RT	0.148*	0.053*	9.18*	− 0.239*	0.008	9.07	− 0.101
P3 amplitude	0.010	0.053*	2.49	0.240*	0.025	1.99	0.182
Upper alpha	0.027	0.059*	3.40*	− 0.251*	0.017	3.36*	− 0.152

Muscular power, muscular endurance, and non-aerobic fitness are the predictors. The behavioral (Hit, d′, RT) and neuroelectric (P3 amplitude, lower alpha ERD, upper alpha ERD) are the dependent outcome variables. In each regression analysis for each dependent outcome variable, the same demographic variable that was correlated with the dependent outcome variable was entered in Step 1 of both the 2-step and 3-step models. For 2-step models, muscular power, muscular endurance, and non-aerobic fitness were entered into the Step 2. For 3-step models, motor competence was entered into the Step 2, and muscular power, muscular endurance, and non-aerobic fitness were entered into the Step 3. Bolded values with a * denote significance at *p* < 0.05.

#### Motor competence

After controlling for age in the first step, ball skills were unrelated to any neurocognitive variable in the 2-step models, while MC and its coordination and dexterity component showed beneficial associations with behavioral outcomes and upper alpha ERD (see Table 4). All these associations remained after further controlling the NAF in the 3-step models, except that upper alpha ERD was no longer predicted by MC (see Fig. 1D).

**Table 4:** The summary of the 2-step and 3-step hierarchical regression analyses using motor competence or its subcomponent as a predictor.

	Step 1	2-Step model			3-Step model		
		Step 2			Step 3		
	*R*^2^	*△R*^2^	F	*Beta*	*△R*^2^	F	*Beta*
**Coordination and dexterity**							
Hit	0.056*	0.099*	6.70*	0.325*	0.063*	7.77*	0.264*
d′	0.056*	0.034	3.48*	0.191	0.025	2.72	0.167
RT	0.148*	0.090*	**11.41**	− 0.310*	0.068*	8.83*	− 0.274*
Lower alpha	0.025	0.050	2.93	− 0.230	0.038	2.37	− 0.205
Upper alpha	0.027	0.079*	4.32*	− 0.291*	0.057*	3.98*	− 0.252*
**Ball skill**							
Hit	0.056*	0.040	3.88*	0.206	0.007	5.60*	0.094
d′	0.053*	0.030	3.29*	0.178	0.016	2.46	0.141
RT	0.148*	0.043	8.66*	− 0.216	0.019	6.79*	− 0.153
Upper alpha	0.027	0.042	2.67	− 0.211	0.017	2.73*	− 0.143
**Motor competence**							
Hit	0.056*	0.205*	12.94*	0.490*	0.109*	9.87*	0.399*
d′	0.053*	0.111	7.14*	0.360*	0.087*	4.70*	0.356*
RT	0.148*	0.119*	13.28*	− 0.373*	0.073*	9.07*	− 0.326*
P3 amplitude	0.010	0.041	1.99	0.220	0.013	1.99	0.136
Lower alpha	0.025	0.046	2.77	− 0.231	0.025	2.00	− 0.189
Upper alpha	**0.**027	0.079*	4.30*	− 0.304*	0.038	3.36*	− 0.234

Coordination and dexterity, ball skill, and motor competence are the predictors. The behavioral (Hit, d′, RT) and neuroelectric (P3 amplitude, lower alpha ERD, upper alpha ERD) are the dependent outcome variables. In each regression analysis for each dependent outcome variable, the same demographic variable that was correlated with the dependent outcome variable was entered in Step 1 of both the 2-step and 3-step models. For 2-step models, Coordination and dexterity, ball skill, and motor competence were entered into the Step 2. For 3-step models, non-aerobic fitness was entered into the Step 2, and Coordination and dexterity, ball skill, and motor competence were entered into the Step 3. Bolded values with a * denote significance at *p* < 0.05.

## Discussion

The purpose of this study was to examine the associations of muscular (power and endurance), flexibility, and balance components of NAF as well as MC and its sub-components with behavioral performance and underlying neuroelectric brain functioning during an auditory oddball task in preschool children. The results showed that the NAF, MC, and components related to muscular fitness and fine motor skills (i.e., coordination and dexterity, ball skill) each had some beneficial associations with behavioral or neuroelectric outcomes. Specifically, muscular endurance showed a unique positive contribution to P3 amplitude, while higher levels of MC and its coordination and dexterity component were associated with superior task performance independent of NAF, with coordination and dexterity showing an additional association with greater upper alpha ERD. Collectively, findings in the current study demonstrated that NAF and MC may have differential associations with cognitive performance and its neuroelectric correlates of temporally different attentional processes during the preschool years.

Although the auditory oddball paradigm is a discrimination task that has been primarily used as a P3-eliciting task in young children^35^, increased hit rate and decreased RT during this task may reflect superior two-choice decision-making process relying on the stimulus categorization and response selection. The positive associations of cognitive performance during this task with NAF and MC were similar to previous research showing beneficial associations of physical fitness^10,14^ and MC^24,56^ with cognitive performance in preschool children. More importantly, MC and its coordination and dexterity component explained a 6–10% of variance in task performance independent of NAF, suggesting their unique contributions to task performance requiring controlled attention in preschool children. It should be noted that although the use of computerized task presumably minimized the potential confounding effect of individual difference in motor time on the task performance indices during the current oddball task, young children who had more developed motor skills, especially the coordination and dexterity, could have generated faster responses because of a shorter time difference between the initiation and the end of a button press. Therefore, the positive associations of MC and coordination and dexterity with task performance observed herein could also attribute to the positive relation of MC and coordination and dexterity with the motor component of response times. This speculation on the particular importance of MC to performance during a psychomotor demanding task was also supported by the lack of findings in the 3-step model analysis using NAF and its subcomponent as a predictor. That is, not only a relatively smaller portion of variance in performance during a psychomotor demanding task was explained by NAF components in the current results as well as aerobic fitness in previously reported preschool^10^ and preadolescent children^37,57^, these associations were significantly attenuated after controlling for MC in the current study. These findings are in line with the recent framework that developing MC, particularly fine motor skills that support coordination and dexterity, may have beneficial association with cognitive function in young children^58^.

Beyond the behavioral outcomes, MC, NAF, and muscular endurance were associated with larger P3 amplitude, although such an association only remained for muscular endurance after controlling for confounders in the 2- and 3-step regression analyses. These findings were similar to previous research in older children^41,42^ but novel in that the beneficial association of muscular endurance with the neural inhibition process needed to reallocate attentional resources was independent of MC. Because muscular endurance was not related to task performance in the 3-step regression analysis, P3 amplitude may manifest a neuroelectric mechanism that is more sensitive than task performance outcomes to capture the association of muscular endurance with attention during the preschool years. Increased P3 amplitude has been theorized to reflect a more mature neuroelectric profile^35^ related to the functioning of fronto-parietal network^31–33^, which is important for the attention network systems^2^. However, NAF and its subcomponents were not correlated with the latency of P3, consisting with a recent systematic review that physical activity and aerobic fitness may have less robust associations with P3 latency throughout the life span^44^ as well as an observational study showing selective association of aerobic fitness with amplitude but not latency measure of P3 in preschool children^12^. Accordingly, muscular endurance may have unique positive associations with brain function underlying attention networks that support task-related attentional allocation processes but not the speed of information processing during early childhood. Alternatively, given the similarity of assessment for muscular endurance and aerobic fitness as well as the correlation between these fitness components in young children, the current findings in the association of muscular endurance with P3 amplitude could also reflect the previously reported association between aerobic fitness with P3 amplitude^12^.

Despite the lack of associations with the amplitude and latency measures of P3, increased MC, ball skills, and coordination and dexterity were correlated with greater ERD in the lower and/or upper alpha frequencies. None of these associations with lower alpha ERD remained after controlling for age and/or NAF, even though the target-elicited ERD appeared more prominent in the lower alpha frequency (see Fig. 1B), likely due to the less complicated attention processes involved in the current oddball task. However, upper alpha ERD, which reflects more task specific cognitive process (i.e., discriminating targets from non-targets in the oddball task), despite being less prominent, was associated with the coordination and dexterity component even after controlling for age and NAF. Given that alpha ERD has been theorized as an indication of cortical activation in facilitating the release from neural inhibition^51^, the observed alpha ERD temporally following P3 (i.e., 630 ms) and behavioral responses (i.e., 589 ms) may reflect a late resetting process that involved strategic attentional updating of mental representation in preparation for the next testing trial^50^. Such a selective association in the upper alpha band suggests that coordination and dexterity were less sensitive to general anticipation of auditory stimuli but beneficial for task-specific processes^49^, namely the preparatory process of discriminating target from standard auditory stimuli. The speculation that coordination and dexterity may be associated with this temporally selective process (i.e., preparatory) was confirmed by the weak to no alpha ERS/ERD during the early stage of stimulus evaluation in the current and previous research^50^. Moreover, parietal upper alpha ERD has been associated with the fronto-parieto-occipital attention system^52–54^, which largely overlaps with the brain structure and connectivity that benefit from MC^59,60^ as well as the attention networks during early development^2^. Thus, the observed association of coordination and dexterity with upper alpha ERD may be a manifestation of superior functioning of these networks.

The differential associations of muscular endurance and coordination and dexterity with neurocognitive indices may suggest different roles these physical ability components play during preschool years. This speculation was partially supported by a recent framework that proposed quantitative and qualitative adaptations as distinguishable mechanisms underlying neurocognitive benefits of physical activity^58^. Our findings that muscular endurance, likely developed through repeated exercise requiring greater metabolic demand, had a positive association with P3 amplitude that reflects the quantitative capacity for processing behaviorally relevant target stimuli^61^. On the other hand, coordination and dexterity, likely developed during the processes of acquiring fine motor skill, had a positive association with cortical activation (i.e., greater alpha ERD) related to qualitative strategy for attentional resetting in preparation for subsequent target events. Given that these associations were absent for other NAF domains reflecting adaptations to less metabolic demand and motor components involving less fine motor skills, the current findings support the notion that increasing metabolic demand and complex fine movements during physical activity may be essential for accruing the largest cognitive benefits^58^. Future research using longitudinal and/or interventional designs is needed to confirm our speculation that early childhood development of physical abilities resulting from qualitatively/quantitatively different adaptations have benefits to differential aspect of neurocognitive function.

The current study is novel in that it is the first to use neuroelectric indices to examine the associations of physical abilities and their sub-components with neurocognitive function in preschool children. Specifically, we focused on NAF domains and MC, which are important for many developmental and health outcomes^20,45^, yet less explored in preschoolers. Moreover, P3 and alpha ERD were used to index different cognitive processes during and following stimulus evaluation as well as a neuroelectric manifestation of brain functioning. Despite these strengths, the conclusions drawn from this study were limited because of its cross-sectional study design and inability to rule out the possibility that better neurocognitive function led to higher levels of NAF and MC. To test the directionality of these relationships, longitudinal research measuring these developmental outcomes at multiple time points is needed. Secondly, IQ, body composition (i.e., body mass index [BMI]), and socioeconomic status, which have been identified as potential confounding factors of early neurocognitive maturation^62,63^, were not available as the controlling variables in the current analyses. Although the current study estimated IQ using a creativity task, this proximity measure only explained limited variance in preschooler’s IQ^64^. Lastly, this study did not include a measure of aerobic fitness because of the focus on the unexplored associations of NAF and MC with neurocognitive function in preschool children, despite previously found correlations of aerobic fitness with NAF, MC, and neurocognitive function during maturation^7,57,65^. Thus, the current findings should be interpreted with caution in comparison with studies measuring all or different aspects of physical fitness. Clearly, future research measuring aforementioned confounders is needed to better isolate the contribution of physical fitness, MC, and their sub-components to neurocognitive function in preschool children.

In conclusion, the current study showed that early childhood muscular endurance was positively related to P3 amplitude. Higher levels of MC and its coordination and dexterity component were related to superior attention performance, with coordination and dexterity having additional association with greater upper alpha ERD. These findings suggest that muscular endurance and coordination and dexterity during preschool years may play roles to effective neural inhibition in facilitating attentional allocation during stimulus evaluation, increased cortical activation in preparation for subsequent stimulus discrimination, as well as superior brain function underlying attention networks. Such findings have considerable contribution to the field as they (1) address the knowledge gap in the relationship between physical activity and cognitive health in children < 5 years old raised by the Committee Scientific Report of the 2018 Physical Activity Guidelines, (2) guide future research to use different neuroelectric indices to better understand the associations of early childhood fitness and MC with temporally different attentional processes, and (3) inform future hypothesis testing on the development (i.e., longitudinal associations) of early childhood fitness and MC with behavioral and neural outcomes of attention. Beyond the scientific significance, the current findings are practically relevant as they can be translated to guide the refinement of currently predominant aerobic exercise programs by supplementing activities designed to improve NAF and MC. Through demonstrating the associations of diverse aspects of physical abilities with behavioral and neuroelectric correlates of cognitive performance in young children, the current study offers a new insight into the potential of exercise as a strategy to enhance neurocognitive correlates of cognitive performance that underlies a host of developmental outcomes such as on-task behaviors, learning, and academic achievement.

## Method

### Participants

Ninety-two (mean age = 68.5 ± 4.7 months, 36% female) typically developed preschool children were recruited from five preschool classrooms affiliated with two different elementary schools located in Tien-Mu area, an area consisting of families mainly from higher socioeconomical background, in Taipei, Taiwan. This convenient sample was chosen because these schools participated in the city-wise preschool fitness assessment program and their principals were interested in additional evaluation on cognitive and brain function. Participants’ legal guardians completed a parent-reported screening questionnaire to indicate that their child had normal, or corrected-to-normal, vision and hearing, and was right-handed, free of cardiovascular diseases (i.e., heart disease, diabetes), neurological diseases (i.e., attention-deficit hyperactivity disorder), and head injuries (i.e., concussion history, loss of consciousness). Eligible participants who passed the screening and their legal guardians provided written informed assent/consent approved by the Institutional Review Board at Taipei Physical Education College and were invited to participate in two testing sessions. All testing procedures involved during these two sessions were performed in accordance with relevant guidelines and regulations. This convenient sample afforded a power > 0.92 to detect a small-to-medium size of the association of one fitness or MC predictor with behavioral (*f*^2^ = 0.16)^14^ and neuroelectric (*r* = 0.37)^12^ cognitive outcomes.

### Procedure

Child participants were instructed to refrain from stimulating activities (i.e., vigorous physical activity) and drinks/foods (i.e., caffeinated snacks) on the day prior to testing on two separate weekdays at their schools. *Day 1* Participants and their legal guardians (if not available, a teacher would accompany) were escorted to a quiet, sound attenuated classroom, followed by detailed explanation of the purpose of this study. Participants who were eligible for participating in this study were then fitted with an EEG cap (~ 15 min). Task-related EEG was then recorded while participants performed an auditory oddball task. *Day 2* Participants completed the Torrance’s Thinking Creatively in Action and Movement (TCAM) test to evaluate creative thinking^66^ as a proxy for estimating intelligence quotient (IQ) in preschool children^64^. Next, participants completed two test batteries to measure MC and NAF. All Day 1 and Day 2 testing occurred on the same day of the week in the morning session that was originally scheduled for fitness classes. Upon completion of each testing day, participants were given small gifts (i.e., stickers, pencils) as rewards.

### Motor competence test

MC was assessed using nine sub-tests from the Basic Motor Ability Test-Revised (BMAT, see supplement 1 for details)^67^, including bead stringing, target throwing, marble transfer, ball striking, basketball throwing, ball kicking, static balance, prone-stand-turn, and 4-cone shuttle run. Each sub-measure was converted into standardized z-scores (speed-related measures were multiplied by − 1). The nine scores were summed to make a composite score of MC (intraclass correlation coefficients [ICC] = 0.93, suggesting excellent reliability])^67^. To explore the associations of different components of MC with cognitive outcomes, three sub-component scores were created based on categories existing in other MC test such as the Movement Assessment Battery for Children^68^. The three sub-components included Coordination and Dexterity (bead stringing, target throwing, marble transfer) as a measure of fine motor control, Ball Skills (ball striking, basketball throwing, ball kicking) as a measure of both fine and gross motor control, and Agility and Balance (static balance, prone-stand-turn, 10-m shuttle run) as a measure of gross motor control^68^.

### Physical fitness test

A battery of fitness testing (see supplement 1 for details) consisting of 60-s crunch curl-ups, standing long jump, single-leg standing on a beam with eyes closed, and sit-and-reach were used to evaluate muscle endurance (mean ICC range: 0.61–0.91), muscle power (mean ICC range: = 0.57–0.90), balance (mean ICC range: = 0.43–0.77), and flexibility (mean ICC range: = 0.74–0.93), respectively^69,70^. These tests were administered according to the guidelines of the Preschool Children Fitness Program, which was developed by the Department of Education of the Taipei City Government to evaluate the developmental trajectories of different fitness domains in preschool children. A fitness composite score was calculated by summing the standardized scores from the four sub-tests^14,16^.

### Cognitive task

The present study employed an auditory oddball task that has been widely used to elicit P3 in young children^35^. The auditory modality of stimulus presentation was chosen because of its greater sensitivity to early childhood brain function and development^35^. The task was administered using Neuroscan Stim software 2.0 (NeuroScan; Charlotte, NC, USA) installed on a laptop with a binaural speaker. The task consisted of 190 standard stimuli (1000 Hz tone) and 60 target stimuli (2000 Hz tone), with each stimulus presenting for 60 ms at 70 dB. The task was divided into 5 blocks of 50 trials (38 standard and 12 target). Within each task block, the two stimulus types were presented in random order using an inter-stimulus interval randomized between 1600 and 1800 ms. A short break (~ 30 s) was provided between blocks and the entire task lasted about 10 min. Prior to the actual testing, participants were verbally explained the task and completed five practice trials. Participants were instructed to respond to the target stimuli by pressing a button as quickly as possible while not responding to the standard stimuli. Despite the emphasis on the simple discriminating processes between target and standard stimuli, omitting the standard stimuli while quickly responding to target stimuli required controlled attention (i.e., inhibit response to standard stimuli, inhibit the biased response selection). Thus, behavioral outcomes such as hit rate and median response time (RT) for target stimuli as well as false alarm rate (FA) and *d*-prime (*d*′) were analyzed.

### Neuroelectric assessment

EEG was measured from a 32-electrode Neuroscan Quik-Cap arranged using the International 10–20 standard (Compumedics, Charlotte, NC). Recordings were referenced to averaged mastoids, with the mid-frontal site (FPz) serving as the ground electrode, and impedance < 10 kΩ. Additional electrodes were placed above and below the left orbit and on the outer canthus of each eye to monitor vertical (VEOG) and horizontal electro-oculographic activity with a bipolar recording. Continuous data were digitized at a sampling rate of 500 Hz, with a DC to 70 Hz filter, and a 60 Hz notch filter using a Neuroscan amplifier (Compumedics, Charlotte, NC). Matlab (R2014a, Mathworks Inc.), EEGLAB toolbox (version 13.4.4)^71^, and ERPLAB toolbox (version 4.0.2.3)^72^ were used for offline data processing. Raw EEG data collected during the cognitive task were corrected for eye-movement artifacts using an independent component analyses (ICA) followed by an autocorrelation procedure rejecting ICA components (i.e., EEG.icaact matrix generated by the ICA procedure) that had a correlation coefficient greater than 0.3 with the raw VEOG time-series data^73^. Corrected data underwent two different processing pipelines to evaluate task-related P3-ERP and event-related de/synchronization (ERS/ERD) in frequency bands.

#### ERP

Epochs were created from − 100 to 1000 ms around the stimulus, baseline-corrected using the − 100 to 0 ms pre-stimulus period, and filtered using a zero phase-shift low-pass filter (IIR Butterworth filter) at 30 Hz (24 dB/oct). Following rejection of epochs corresponding with response errors or identified artifacts exceeded ± 100 uV, remaining epochs were averaged to construct ERPs. The P3 component during the target trials was quantified by the peak latency and mean amplitude within 50 ms of the largest positive peak within a 300–800 ms post-stimulus window. The amplitude and latency of P3 were quantified at midline electrodes (i.e., Fz, Cz, Pz) to allow confirmatory analysis on the parietally centered P3^35^ and its use in the subsequent statistical analysis^50^.

#### ERS/ERD

Epochs were created from − 2000 to 2000 ms time-locked to stimulus onset. Trials corresponding to a response error and containing artifacts with amplitudes exceeding ± 150 μV were discarded. Oscillatory EEG power was computed by a continuous Morlet wavelet transform (wavelet factor = 6) of single-trial data for the frequency band ranging from 1 to 15 Hz using the *newtimef()* function. Oscillatory power, defined as the square of the modulus of the resulting complex number, was averaged across trials, rescaled by the baseline values from − 300 to − 100 ms relative to stimulus onset, and a log10 transform of this quotient was applied (dB power = 10 × log10 [power/baseline]), which allowed direct comparison of results across frequencies. The study focused on lower alpha (8–10 Hz) and upper alpha (10–12 Hz) at Pz as these frequency bands at the parietal region are correlated with P3 and cognitive performance^49,50^. According to the visual inspection on Fig. 1B, the 700–1000 ms time window following stimulus onset was used to calculate the average alpha ERD.

### Statistical analysis

Data were analyzed using SPSS (SPSS v. 22, Chicago, IL) with the family-wise alpha threshold for all tests set at *p* = 0.05. To confirm Pz as the region of interest to examine P3 (amplitude and latency) and alpha ERD (lower alpha and lower alpha) measures during the attentional processes in response to target stimuli, a two-way 2 (Stimulus: Target, Standard) × 3 (Electrode: Fz, Cz, Pz) repeated measure ANOVA was conducted to verify the target-specific and parietal-centered P3 and alpha ERD. A bivariate correlation was performed to identify the associations of demographic factors and cognitive outcomes with NAF, MC, and their sub-components. Next, NAF, MC, and their sub-components that were significantly correlated with any behavioral and neuroelectric outcomes in the bivariate correlation analyses were used as predictors to perform separate hierarchical regression analyses. In each of these separate analyses, the behavioral and neuroelectric measures obtained from the oddball task were used as the dependent outcome variables. Of these hierarchical regression analyses, a series of 2-step models were used to determine the association of each predictor with the cognitive outcomes in each regression analysis independent of demographic variables. To achieve this, demographic variables (i.e., age) that were correlated with the predictor or the behavioral and neuroelectric cognitive outcome variable were entered into the first step and the predictor was entered into the second step.

Additional 3-step hierarchical regressions analyses were performed. In each of these 3-step models, demographic variables (i.e., age) that were correlated with the predictor or the behavioral and neuroelectric cognitive outcome variable were entered into the first step, MC was entered into the second step, and the NAF-related predictor was entered into the third step. Similar 3-step regression analyses were conducted using NAF as the controlling variable in the second step and MC and its sub-components as predictors in the third step. The goal of these 3-step regression analyses was to further determine (1) the unique contribution of each NAF-related predictor to the behavioral and neuroelectric cognitive outcomes after controlling for MC as well as (2) the unique contribution of MC and its sub-components to the behavioral and neuroelectric cognitive outcomes after controlling for NAF.

## Supplementary Information

Supplementary Information.

## Data Availability

The datasets generated during and/or analyzed during the current study are available from the corresponding author on reasonable request.
