# Supplementary material for: The relationship of muscular endurance and coordination and dexterity with behavioral and neuroelectric indices of attention in preschool children
Source: Sci Rep. 2022 Apr 29;12:7059. doi: 10.1038/s41598-022-11161-4 (PMC9054790; doi:10.1038/s41598-022-11161-4)
Supplement: Supplementary file 1 — Supplementary Information. [file 41598_2022_11161_MOESM1_ESM.docx]

**Motor competence test**

Motor competence was assessed using nine sub-tests from the Basic Motor Ability Test-Revised (BMAT) (1), including (1) Bead Stringing (Number of beads strung within 40s. One attempt), (2) Target Throwing (Summed scores of throwing a bean beg inside a 36 cm height bucket with a 33 cm diameter placed on the ground with a distance of 90, 180, 270, 360, and 450 cm. Two attempts at each distance for each hand. A hit inside the bucket scored 2 points and a hit on the bucket scored 1 point), (3) Marble Transfer (Number of marbles transferred from one container to the other container that was 30 cm apart within 20s. Summed over attempts, with one for each hand), (4) Ball Striking (Summed scores of striking ball with a diameter of 25 cm by hand at the waist height while standing at a line 120 cm from the target, which consisted of three horizontally concatenated 240 cm height x 60 cm width rectangles on the wall. Five attempts for each hand. A hit in the middle rectangle scored 4 points and a hit in the flanking rectangles scored 2 points), (5) Basketball Throwing (Maximum distance in centimeters selected from 3 attempts of throwing using both hands from chest), (6) Ball Kicking (Summed scores of kicking ball with a diameter of 25 cm while standing at a line 5 meters from the target, which consisted of three horizontally concatenated 240 cm height x 40 cm width rectangles on the wall. Five attempts for each leg. A hit in the middle rectangle scored 4 points and a hit in the flanking rectangles scored 2 points), (7) Static Balance (Time averaged across four attempts of single leg standing. Two attempts for each leg, with one attempt having eyes closed and the other having eyes opened), (8) Prone-Stand-Turn (Repetition of standing up from a prone position, touching an object at the participant’s height, and returning to the prone position within 20 seconds. One attempt), and (9) 4-cone shuttle run (Time completing a shuttle run between the starting line and each of the four cones placed at a distance of 150, 300, 450, 600 cm. One attempt). Each of the nine sub-measure was converted into standardized z-scores (speed-related measures were multiplied by -1) and then summed together to make a composite score of motor competence. This composite score has been demonstrated to afford the examination on the development of motor competence in response to interventions aimed to promote motor skills (2) and on the association of motor competence with childhood cognition and its underlying neuroelectric function (3). The test-retest reliability for the BMAT was high (intraclass correlation coefficients [ICC] = 0.93, suggesting excellent reliability) (1). Three sub-component scores were created based on categories existing in other MC test such as the Movement Assessment Battery for Children (68). The three sub-components included Coordination and Dexterity (bead stringing, target throwing, marble transfer) as a measure of fine motor control, Ball Skills (ball striking, basketball throwing, ball kicking) as a measure of both fine and gross motor control, and Agility and Balance (static balance, prone-stand-turn, 10-m shuttle run) as a measure of gross motor control (4).

**Physical fitness test**

A battery of fitness testing consisting of crunch curl-ups (one attempt of maximum repetition within 60 seconds), standing long jump (maximum distance in centimeters selected from 3 attempts), single-leg standing on a beam (height = 3 cm, width = 3 cm, length = 30 cm) with eyes closed (maximum duration averaged across two attempts. One attempt for each leg), and sit-and-reach (maximum distance selected from 3 attempts) were used to evaluate muscle endurance (ICC = 0.61-0.91), muscle power (ICC = 0.57-0.90), balance (ICC = 0.43-0.77), and flexibility (ICC = 0.74-0.93), respectively (5, 6). These tests were administered according to the guidelines of the Preschool Children Fitness Program (PCFP), which was specifically developed by the Department of Education of the Taipei City Government to evaluate the developmental trajectories of different fitness domains in preschool children. A fitness composite score was calculated by summing the standardized scores from the four sub-tests (2, 7).

**Table S1.** The description of tests used to measure different sub-components of motor competence and non-aerobic fitness

| **Measure** | **Task Description** |
| --- | --- |
| Motor Competence: Sum of all nine sub-tests from the Basic Motor Ability Test | |
| Coordination and Dexterity: Index of fine motor skill. Sum of Bead stringing, Target throwing, and Marble transfer. | Bead Stringing:   - Maximum number of beads strung within 40s from one attempt |
|  | Target Throwing   - Summed scores of throwing a bean beg inside a 36 cm height bucket with a 33 cm diameter placed on the ground with a distance of 90, 180, 270, 360, and 450 cm - A hit inside the bucket scored 2 points and a hit on the bucket scored 1 point - Two attempts at each distance for each hand |
|  | Marble Transfer   - Summered number of marbles transferred from one container to the other container that was 30 cm apart within 20s - One attempt for each hand |
| Ball Skills: Index of fine and gross motor skill. Sum of Ball striking, Basketball throwing, and Ball kicking. | Ball Striking   - Summed scores of striking ball with a diameter of 25 cm by hand at the waist height while standing at a line 120 cm from the target, which consisted of three horizontally concatenated 240 cm height x 60 cm width rectangles on the wall - A hit in the middle rectangle scored 4 points and a hit in the flanking rectangles scored 2 points - Five attempts for each hand |
|  | Basketball Throwing   - Maximum distance in centimeters selected from 3 attempts of throwing using both hands from chest |
|  | Ball Kicking   - Summed scores of kicking ball with a diameter of 25 cm while standing at a line 5 meters from the target, which consisted of three horizontally concatenated 240 cm height x 40 cm width rectangles on the wall - A hit in the middle rectangle scored 4 points and a hit in the flanking rectangles scored 2 points Five attempts for each leg |
| Agility and Balance: Index of gross motor skill. Sum of Static balance, Prone-stand-turn, and 4-cone shuttle run. | Static Balance   - Time averaged across four attempts of single leg standing - Two attempts for each leg - Each leg had one attempt with eyes closed and one attempt with eyes opened |
|  | Prone-stand-turn   - Maximum repetition of standing up from a prone position, touching an object at the participant’s height, and returning to the prone position within 20 seconds from one attempt |
|  | 4-cone shuttle run   - Time in second for completing a shuttle run between the starting line and each of the four cones placed at a distance of 150, 300, 450, 600 cm from one attempt |
| Non-Aerobic Fitness: Composite score calculated by summing the standardized scores from four sub-tests of the Preschool Children Fitness Program | |
| Muscular Power | Maximum distance in centimeters selected from 3 attempts of standing long jump |
| Muscular Endurance | Maximum repetition of crunch curl-ups within 60 seconds from one attempt |
| Balance | Maximum duration in second averaged across one attempt of single left leg standing on a beam with eyes closed and one attempt of single right leg standing on a beam with eyes closed |
| Flexibility | Maximum distance selected from 3 attempts of sit-and-reach |

**Confirmation analysis on parietal P3 and alpha ERD**

To confirm Pz as the region of interest to examine P3 and alpha ERD during the attentional processes in response to target stimuli, a two-way 2 (Stimulus: Target, Standard) x 3 (Electrode: Fz, Cz, Pz) repeated measure ANOVA was conducted to verify the target-specific and parietal-centered P3 and alpha ERD.

Based on our visual inspection, a clear target P3 was observed at Pz (Figure 1.A and 1.C). This observation was confirmed by an Electrode main effect on amplitude, *F* = 137, *p* < .001, η_p_^2^ = .647, with Pz (9.7 ± 0.7 uv) showing significantly larger amplitude compared with Cz (7.2 ± 0.5 uv) and Fz (1.5 ± 0.6 uv), *p*s < .001, as well as a Stimulus main effect, *F* = 129, *p* < .001, η_p_^2^ = .633, with target stimulus (10.7 ± 0.8 uv) eliciting larger amplitude compared with standard stimuli (1.4 ± 0.4 uv). These main effects were further superseded by a Stimulus x Electrode interaction, *F* = 38.2, *p* < .001, η_p_^2^ = .338, showing that the increased amplitude for target compared to standard stimuli at Pz (12.0 ± 1.0 uv) and Cz (10.6 ± 0.9 uv) were significantly greater compared to Fz (5.6 ± 0.8 uv), *p*s < .001. Although analysis on latency showed non-significant Stimulus x Electrode interaction, *F* = 0.7, *p* = .391, η_p_^2^ = .010, and Stimulus main effect, *F* = 2.4, *p* = .101, η_p_^2^ = .030, a significant Electrode main effect was observed, *F* = 5.2, *p* = .009, η_p_^2^ = .065, with Pz (630 ± 11 ms) showing significantly shorter latency compared to Fz (666 ± 11 ms) and with Cz (647 ± 11 ms) showing no difference compared to Fz and Pz.

Based on our visual inspection on the time-frequency plot (Figure 1.B and 1.C), alpha ERS was relatively weak at the upper alpha band and absent at the lower alpha band. Lower and upper alpha ERS at Pz between 200-500ms following the onset of target stimulus were calculated to explore their associations with task performance, non-aerobic fitness, motor, and their sub-component scores but no significant correlation was found (|*r*s| < 0.169, *p*s > 0.145). This exploratory analysis was conducted to (1) eliminate the possibility that non-aerobic fitness and motor competence have associations with alpha ERS/ERD during the stimulus evaluation prior to the response execution and (2) inform the temporal specificity of alpha ERD during a later time window following response execution.

In contrast, a clear alpha ERD was observed at the Pz electrode between 700-1000 ms after stimulus onset. This time window was used to calculate the averaged values in lower and upper alpha frequency bands. The selection of the time-frequency representations of interest at Pz was confirmed by an Electrode main effect on both lower and upper alpha ERD (i.e., decreased power), *F*s > 9.8, *p*s < .001, η_p_^2^ > .116, with Pz (lower: -0.778 ± 0.09 db; upper: -0.39 ± 0.10 db) showing greater ERD compared with Cz (lower: -0.466 ± 0.08 db; upper: -0.23 ± 0.09 db) and Fz (lower: -0.221 ± 0.08 db; upper: -0.13 ± 0.08 db), *p*s < .001, as well as a Stimulus main effect, *F*s > 12, *p*s < .001, η_p_^2^ = .138, with target (lower: -1.058 ± 0.147 db; upper: -0.518 ± 0.15 db) stimulus inducing greater ERD compared with standard (lower: 0.081 ± 0.043 db ;upper: 0.02 ± 0.05 db) stimuli. These main effects were further superseded by a Stimulus x Electrode interaction, *F*s < 12.8, *p*s < .001, η_p_^2^ < .146, showing that the increased ERD from standard to target stimuli at Pz (lower: -1.786 ± 0.184 db; upper: -0.925 ± 0.193 db) was significantly greater compared to Cz (lower: -1.095 ± 0.171 db; upper: -0.457 ± 0.164 db) and Fz (lower: -0.537 ± 0.160 db; upper: -0.234 ± 0.162 db), *p*s < .001. Taken together, the observed P3 and alpha ERD specifically following the target stimulus at Pz electrode site replicated previous findings in older children and adults (8, 9), confirming the adequacy of the P3 and alpha ERD measures at Pz for subsequent analysis.

Based on the visual inspection of the Figure 3.C, alpha ERD induced by target stimulus during the oddball task was not restricted in the midline electrode. Therefore, an additional set of analysis was conducted based on a region of interest (ROI) averaging across CP3, CPZ, CP4, P3, PZ, and P4. As shown in Table S2, there were some bivariate correlations between non-aerobic fitness, muscular power, motor competence, coordination and dexterity, lower alpha ERD, and upper alpha ERD. These variables were used to conduct the subsequent hierarchical regression analyses using 2-step and 3-step models (see Table S3 and Table S4). Similar to the analysis using only PZ electrode, non-aerobic fitness and its muscular power component were not associated with lower or upper alpha ERD after controlling for age in the 2-step models as well as after controlling for both age and motor competence in the 3-step models. Motor competence was associated with upper alpha ERD after controlling for age in the 2-step model, but such an association became non-significant after controlling for non-aerobic fitness. Coordination and dexterity was associated with both lower and upper alpha ERD after controlling for age in the 2-step models. After further controlling for non-aerobic fitness, only the association with upper alpha ERD remained significant. Taken together, the ROI-based analysis generated a pattern of findings that are similar to the PZ-based analysis reported in the current study.

**Table S2.** The correlation matrix showing correlations (*r* coefficients) of demographic variables with non-aerobic fitness, motor competence, and ROI-based alpha ERD outcomes.

|  | **Lower Alpha ERD** | **Upper Alpha ERD** |
| --- | --- | --- |
| **Age** | -0.212 | -0.184 |
| **Sex** | -0.045 | 0.096 |
| **Creativity** | -0.02 | 0.037 |
| **Non-aerobic fitness** | **-.229*** | **-.252*** |
| **Motor competence** | **-.283*** | **-.329*** |
| **Muscular power** | **-.239*** | -0.223 |
| **Muscular endurance** | -0.207 | -0.178 |
| **Flexibility** | -0.051 | -0.034 |
| **Balance** | 0.072 | -0.053 |
| **Coordination and Dexterity** | **-.277**** | **-.332**** |
| **Ball Skills** | -0.188 | -0.224 |
| **Agility and Balance** | 0.031 | 0.104 |

Bold values with a * denote significant correlation at *p* < 0.05.

**Table S3.** The summary of the 2-step and 3-step hierarchical regression analyses on ROI-based alpha ERD outcomes using non-aerobic fitness or its subcomponent as a predictor

|  |  |  | ***2-Step Model*** | | |  | ***3-Step Model*** | | |
| --- | --- | --- | --- | --- | --- | --- | --- | --- | --- |
|  | ***Step 1*** |  | ***Step 2*** | | |  | ***Step 3*** | | |
|  | ***R^2^*** |  | ***△R^2^*** | **F** | ***Beta*** |  | ***△R^2^*** | **F** | ***Beta*** |
| **Muscular Power** | | | | | | | | | |
| Lower Alpha | .045 |  | .033 | 3.09 | -0.192 |  | .032 | **3.60*** | -0.189 |
| **Non-Aerobic Fitness** | | | | | | | | | |
| Lower Alpha | .045 |  | .032 | 3.03 | -0.185 |  | .019 | **3.97*** | -0.145 |
| Upper Alpha | .034 |  | .044 | 3.08 | -0.218 |  | .024 | **4.08*** | -0.165 |

Muscular power and non-aerobic fitness are the predictors. The lower alpha ERD and upper alpha ERD are the dependent outcome variables. In each regression analysis for each dependent outcome variable, age was entered in Step 1 of both the 2-step and 3-step models. For 2-step models, muscular power and non-aerobic fitness were entered into the Step 2. For 3-step models, motor competence was entered into the Step2, and muscular power or non-aerobic fitness were entered into the Step 3. Bolded values with a * denote significance at *p* < 0.05.

**Table S4.** The summary of the 2-step and 3-step hierarchical regression analyses on ROI-based alpha ERD outcomes using motor competence or its subcomponent as a predictor

|  |  |  | ***2-Step Model*** | | |  | ***3-Step Model*** | | |
| --- | --- | --- | --- | --- | --- | --- | --- | --- | --- |
|  | ***Step 1*** |  | ***Step 2*** | | |  | ***Step 3*** | | |
|  | ***R^2^*** |  | ***△R^2^*** | **F** | ***Beta*** |  | ***△R^2^*** | **F** | ***Beta*** |
| **Coordination and Dexterity** | | | | | | | | | |
| Lower Alpha | .041 |  | **.053*** | **3.97*** | **-0.239*** |  | .040 | **3.17*** | -0.211 |
| Upper Alpha | .034 |  | **.087*** | **5.03*** | **-0.305*** |  | **.067*** | **4.08*** | **-0.273*** |
| **Motor Competence** | | | | | | | | | |
| Lower Alpha | .045 |  | .048 | 3.73 | -0.237 |  | .024 | 2.70 | -0.188 |
| Upper Alpha | **.**034 |  | **.078*** | **4.61*** | **-0.303*** |  | .044 | **3.32*** | -0.252 |

Coordination and dexterity and motor competence are the predictors. The lower alpha ERD and upper alpha ERD are the dependent outcome variables. In each regression analysis for each dependent outcome variable, age was entered in Step 1 of both the 2-step and 3-step models. For 2-step models, Coordination and dexterity and motor competence were entered into the Step 2. For 3-step models, non-aerobic fitness was entered into the Step2, and Coordination and dexterity and motor competence were entered into the Step 3. Bolded values with a * denote significance at *p* < 0.05.

**Comparison of analysis results with and without participants who had missing creativity scores**

Creativity scores from three participants were missing. Comparison on results of bivariate correlation between all variables using the sample with (mean-replaced creativity) and without these three participants showed no difference. Therefore, creativity score was not used in any of the 2-step and 3-step model regression analyses and had no influences on the main findings presented in the current study. The only exceptions are the creativity scores in Table 1 and the creativity-related correlation coefficients in Table 2, as these values were obtained using the sub-sample of 73 participants.

**Reference**

1. Arnheim D, Sinclair W, Sinclair S. The basic motor ability tests-revised. The Clumsy Child. 1979:119-44.

2. Chang YK, Tsai YJ, Chen TT, Hung TM. The impacts of coordinative exercise on executive function in kindergarten children: An ERP study. Experimental Brain Research. 2013;225(2):187-96.

3. Hung C-L, Chang Y-K, Chan Y-S, Shih C-H, Huang C-J, Hung T-M. Motor ability and inhibitory processes in children with ADHD: a neuroelectric study. J Sport Exerc Psychol. 2013;35(3):322-8.

4. Pitcher TM, Piek JP, Hay DA. Fine and gross motor ability in males with ADHD. Developmental medicine and child neurology. 2003;45(8):525-35.

5. Oja L, Jürimäe T. Assessment of motor ability of 4‐and 5‐year‐old children. American Journal of Human Biology: The Official Journal of the Human Biology Association. 1997;9(5):659-64.

6. Van Waelvelde H, Peersman W, Lenoir M, Smits Engelsman BC. The reliability of the Movement Assessment Battery for Children for preschool children with mild to moderate motor impairment. Clinical rehabilitation. 2007;21(5):465-70.

7. Tsai Y-J, Huang C-J, Hung C-L, Kao S-C, Lin C-F, Hsieh S-S, et al. Muscular fitness, motor competence, and processing speed in preschool children. European Journal of Developmental Psychology. 2020;17(3):415-31.

8. Raine LB, Kao S-C, Pindus D, Westfall DR, Shigeta TT, Logan N, et al. A large-scale reanalysis of childhood fitness and inhibitory control. Journal of Cognitive Enhancement. 2018;2(2):170-92.

9. Klimesch W. EEG alpha and theta oscillations reflect cognitive and memory performance: a review and analysis. Brain research reviews. 1999;29(2-3):169-95.
